# Strong and Long-Lasting Antinociceptive and Anti-inflammatory Conjugate of Naturally Occurring Oleanolic Acid and Aspirin

**DOI:** 10.3389/fphar.2016.00202

**Published:** 2016-07-12

**Authors:** Barbara Bednarczyk-Cwynar, Natalia Wachowiak, Michal Szulc, Ewa Kamińska, Anna Bogacz, Joanna Bartkowiak-Wieczorek, Lucjusz Zaprutko, Przemyslaw L. Mikolajczak

**Affiliations:** ^1^Department of Organic Chemistry, Faculty of Pharmacy, Poznan University of Medical SciencesPoznan, Poland; ^2^Department of Pharmacology, Poznan University of Medical SciencesPoznan, Poland; ^3^Laboratory of Experimental Pharmacogenetics, Department of Clinical Pharmacy and Biopharmacy, Poznan University of Medical SciencesPoznan, Poland; ^4^Department of Pharmacology and Phytochemistry, Institute of Natural Fibres and Medicinal PlantsPlewiska, Poland

**Keywords:** triterpenes, oleanolic acid, acyloxyimino derivatives, acetylsalicylic acid, anti-inflammatory activity, antinociceptive activity, cytokine levels, mRNA expression

## Abstract

The conjugate **8** was obtained as a result of condensation of 3-hydroxyiminooleanolic acid morfolide **(7)** and aspirin in dioxane. Analgesic effect of OAO-ASA **(8)** for the range of doses 0.3–300.0 mg/kg (*p.o*.) was performed in mice using a *hot-plate* test. Anti-inflammatory activity was assessed on carrageenan-induced paw edema in rats for the same range of doses. The conjugate OAO-ASA **(8)** did not significantly change locomotor activity of mice, therefore sedative properties of the compound should be excluded. The compound **8** proved a simple, proportional, dose-dependent analgesic action and expressed strong anti-inflammatory activity showing a reversed U-shaped, dose-dependent relation with its maximum at 30.0 mg/kg. After its combined administration with morphine (MF, 5.0 mg/kg, *s.c*.) the lowering of antinociceptive activity was found; however, the interaction with naloxone (NL, 3.0 mg/kg, *s.c*.) did not affect the antinociceptive effect of OAO-ASA **(8)**, therefore its opioid mechanism of action should be rather excluded. After combined administration with acetylsalicylic acid (ASA, 300.0 mg/kg, *p.o.*) in *hot-plate* test, the examined compound **8** enhanced the antinociceptive activity in significant way. It also shows that rather the whole molecule is responsible for the antinociceptive and anti-inflammatory effect of the tested compound **8**, however, it cannot be excluded that the summarizing effect is produced by ASA released from the compound **8** and the rest of triterpene derivative. The occurrence of tolerance for triterpenic derivative **8** was not observed, since the analgesic and anti-inflammatory effects after chronic administration of the conjugate OAO-ASA **(8)** was on the same level as after its single treatment. It seemed that the anti-inflammatory mechanism of action of OAO-ASA **(8)** is not simple, even its chronic administration lowered both blood concentration of IL-6 and mRNA IL-6 expression. However, the effects of the conjugate OAO-ASA **(8)** on TNF-α level and mRNA expression were opposite. Moreover, compound **8** did not change unequivocally mRNA TLR1, and TLR3 expression. Concluding, the obtained results regarding the antinociceptive and anti-inflammatory activity of new conjugate of oleanolic acid oxime and acetylsalicylic acid (OAO-ASA **8**) are very interesting, but for explanation of its mechanism of action, more detailed studies are necessary.

## Introduction

Availability and diversity of plant derived therapeutic materials and broad spectrum of their pharmacological activities causes scientists to think hard about looking for not only new chemical compounds included in these plant materials but also to obtaining of semisynthetic derivatives of known compounds with proved pharmacological activity. There are great hopes surrounding triterpenes, a huge group of natural compounds with such broad spectrum of pharmacological activity. One of compounds belonging to this group is oleanolic acid, probably the most widespread triterpene in plant kingdom. Scientists proved its antihypertensive, antiatherosclerotic, antioxidant, anticancer, gastroprotective, hepatoprotective, anti-inflammatory, antidiabetic, and another kinds of pharmacological activities (Liu, 1995; Somova et al., 2003; Dzubak et al., 2006; Sultana and Ata, 2008; Pollier and Goossens, 2012).

There are known some triterpenes, mainly of oleanane, ursane, and lupane groups, that exhibit antinociceptive and anti-inflammatory activity (e.g., Otuki et al., 2005a; Aguirre et al., 2006; Maia et al., 2006b; Saleem, 2009). Promising results were also obtained for α- and β-amyrin. In tests performed upon mice and rats, antinociceptive activity of both amyrins was proved. This activity was probably connected with vanilloid receptors and opioid mechanism (Oliveira et al., 2005). Antinociceptive activity was also proved for oleanolic acid, which also has β-amyrin skeleton. The effectiveness of action of oleanolic acid was also dependent on the amount of dose and the mechanism was also connected with vanilloid and opioid receptors (Maia et al., 2006a).

Oleanolic acid, as many other triterpenes, presents wide range of pharmacological activities along with low toxicity and very good tolerance. Because natural compounds exhibit often relatively low level of biological activity, numerous experiments are performed in order to obtain semisynthetic derivatives with higher therapeutic potential and lower toxicity toward an organism. The biological tests have shown that compounds having a hydroxyimino group within the molecule of triterpene belong to a class of the most active species (Lisiak et al., 2014). Further significant increases of pharmacological activity of triterpenic derivatives can be obtained by their simple modification on the route of hydroxyimino function acylation (Bednarczyk-Cwynar et al., 2012; Kaminskyy et al., 2012). The additional element that increases pharmacological activity of such triterpenic derivative can be the C-17 modified carboxyl function. It is known from many years that conjugate of salicylic acid with morpholine is a effective anti-inflammatory agent (Thieblot et al., 1961) so we decided to obtain an oxime of oleanolic acid in which the =NOH function will be acylated with acetylsalicylic moiety and the C-17 carboxyl group will be transformed into morpholide function.

Still scientists have performed many experiments to obtain new different derivatives of ASA mostly to avoid aspirin-induced gastrointestinal effects including decreasing the acidity and increasing the pharmacological activity of the new designed aspirin-like drugs (Khalikov et al., 2006; Jiang et al., 2010; Huang et al., 2012; Hussain et al., 2013). Recently, the new hydrophobic analogs of aspirin were developed showing inhibition of the production of pro-inflammatory and enrichment of anti-inflammatory cytokines (Kalathil et al., 2016).

In view of the above mentioned possibilities, the main aim of this study was to evaluate an acute and subchronic antinociceptive and anti-inflammatory activity of new ASA and triterpenic derivative, named as conjugate OAO-ASA **(8)**, in animal models. Furthermore, in order to determine the possible contribution of only ASA activity released from the test compound in rodents, the use of ASA in complimentary doses to its presence in OAO-ASA was also performed. Moreover, to evaluate the possible OAO-ASA anti-inflammatory mechanism of action, its interaction with ASA or effect on the concentration or mRNA expression of pro-inflammatory cytokines (IL-6, TNF-α) or toll-like receptors (TLR1, TLR3) were investigated. Moreover, keeping in mind the potential opioid mechanism of action of some oleanolic acid derivatives (Maia et al., 2006b), the interaction of conjugate OAO-ASA **(8)** with morphine and opioid antagonist was assessed.

## Materials and Methods

### Chemicals

All solvents and reagents for synthesis and purification of triterpenic compounds were pure for analysis grade and purchased from Sigma–Aldrich or Chempur. Carrageenan and Tween 80 were purchased from Sigma–Aldrich, USA; morphine sulfate (morphini sulfas 20 mg/ml) was purchased from Polfarma, Poland; acetylsalicylic acid from Galfarma, Cracow, Poland, naloxone hydrochloride (Naloxonum hydrochloricum WZF 0.4 μg/ml) from WZF Polfa S.A., Warsaw, Poland, 0.9% saline solution was purchased from Fresenius Kabi, Poland. Oleanolic acid (3β-hydroxyolean-12-en-28-oic acid) was applied as a parent compound for further chemical transformations. It was isolated by us from a by-product residue obtained during industrial production of mistletoe extract.

### Animals

Experiments were performed on male Swiss mice (20–30 g) and male Wistar rats (250–350 g) housed in controlled room temperature (20 ± 0.2°C) and humidity (65–75%) under a 12 h : 12 h light-dark cycle (lights on 7 a.m.). Animals were kept in groups of 8–10 mice and 4–5 rats in light plastic cages and had a free access to standard laboratory diet (pellets-Labofeed B) and tap water in their cages.

The experiments with animals were performed in accordance with Polish governmental regulations (Dz.U.05.33.289, 2005). The study was conducted in accordance to ethical guidelines for investigations of experimental pain in conscious animals and the study protocol was approved by the Local Ethics Committee of the Use of Laboratory Animals in Poznań (52/2011).

### Instruments

Melting points were measured in open capillaries using the Büchi apparatus. NMR spectra for hydrogen atoms (^1^H) and for carbon atoms (^13^C) were recorded in deuterated chloroform solution using the Varian Gemini 300 VT apparatus, for frequencies 400 MHz and 100 MHz, respectively, with TMS as an internal standard. The values for chemical shifts are given in δ, exact to ±0.01 ppm and ±0.1 ppm, respectively; *J-*values are given exact to ±0.1 Hz. The multiplicities of signals in ^1^H NMR spectra were marked as follows: **s** – singlet, **d** – doublet, **t** – triplet, **dd** – doublet of doublets, **td** – triplet of doublets, **m** – multiplet. Mass spectrum (MS) was recorded using the AMD 402 spectrometer with electroionization. The elucidation of the chemical structures was based on ^1^H NMR, ^13^C NMR, and MS analysis.

Horizontal locomotor activity of mice after application of OAO-ASA **(8)** was evaluated using a licensed activity meter (Activity Cage, Ugo Basile, Italy). Analgesic activity of conjugate **8** was evaluated with hot-plate method using a Hot-Plate Analgesia Meter (Ugo Basile, Italy). Anti-inflammatory activity of OAO-ASA **(8)** was evaluated using a plethysmometer (Hugo Sachs Electronic, Germany).

### Methods

#### TLC Analysis

The thin layer chromatography (TLC) analysis (reactions progress and the level of compounds purity) was conducted on HPTLC aluminum sheets covered with silica gel 60 F245 (Merck) and benzene with ethyl acetate in a volume ratio of 4:1 as an eluent. The chromatograms were visualized by spraying them with 10% ethanolic solution of sulfuric acid and heating them at about 110°C for a few minutes.

#### Oxime of Oleanolic Acid Morpholide (7) Acylation with ASA

ASA (2.16 g, 12 mmol) and *N*,*N*-dicyclohexylcarbodiimide (DCC, 3.09 g, 15 mmol) were added to a stirred at room temperature solution of oxime of oleanolic acid morpholide (**7**, 4.8 g, 10 mmol) in dioxane (100 ml). After substrate total consumption (TLC control) the mixture after reaction was filtered off. The filtrate was poured into a fivefold volume of water acidified with HCl. The obtained white precipitate was filtered off, washed with water, dried and re-precipitated with water from an ethanolic solution.

**3 -(2 -Acetoxy)benzoyloxyiminoolean -12 -en -28 -oic acid morpholide** (OAO-ASA, **8**) C_43_H_60_N_2_O_6_, mol. mass 700.4451, yield: 96.9%, mp.: 131–137°C, white powder. **^1^H NMR**: 7.97 (1H, dd, *J* = 1.7 and 7.5 Hz, CH_3_OCO-**Ar**-COON=C<) and 7.57 (1H, td, *J* = 1.1 and 7.8 Hz, CH_3_OCO-**Ar**-COON=C<) and 7.32 (1H, td, *J* = 1.1 and 7.6 Hz, CH_3_OCO-**Ar**-COON=C<) and 7.13 (1H, dd, *J* = 0.6 and 8.2 Hz, CH_3_OCO-**Ar**-COON=C<), 5.27 (1H, t, *J* = 3.5 Hz, C_12_-H), 3.70–3.58 (8H, m, Mor), 3.08 (1H, d, *J* = 11.4 Hz, C_18_-H_β_), 2.34 (3H, s, **CH_3_**OCO-Ar-COON=C<), 1.33, 1.18, 1.13, 1.04, 0.93, 0.90 and 0.78 (7 × 3H, 7 × s, 7 CH_3_ groups); **^13^C NMR**: 176.3 (C_q_, C-28), 175.1 (C_q_, C-3), 169.6 (C_q_, CH_3_O**CO**-Ar-COON=C<), 162.0 (C_q_, CH_3_OCO-Ar-**CO**ON=C<), 150.6 and 122.8 (2 × C_q_, CH_3_OCO-Ar-COON=C<), 133.7, 131.2, 125.9, and 124.0 (4 × CH, CH_3_OCO-**Ar**-COON=C<), 144.8 (C_q_, C-13), 121.3 (CH, C-12), 66.9 × 2, 46.0 and 41.9 (4 × CH_2_, Mor), 46.2 (C_q_, C-17); 21.0 (CH_3_, **CH_3_**OCO-Ar-COON=C<); **Ar**, aromatic ring; **Mor**, morpholine ring. **MS-EI**: 700.6 (22.9%, M^+^).

#### Analgesic and Anti-inflammatory Activity of OAO-ASA (8)

In the first step, acute toxicity study of OAO-ASA **(8)** was evaluated using orally application for mice, according to OECD TG 420 (for chemical substances, from January 21, 2001).

#### Locomotor Activity Test

The tests were done 60 min after OAO-ASA **(8)** administration which was given in the dose of 0.3, 3.0, 30.0, and 300.0 mg/kg (*p.o.*) dissolved in 5% Tween 80. The data obtained was expressed as signals corresponding to animal movements for 5 min after previous 20 min habituation to the activity meter cage. Next, horizontal locomotor activities of mice was evaluated using the licensed activity meter.

#### Analgesic Activity: Hot-Plate Test

Mice were placed on a hot plate apparatus which was heated to a constant temperature of 52 ± 2°C. A single dose of OAO-ASA **(8)** in the range of 0.3, 3.0, 30.0, and 300.0 mg/kg were given (*p.o.*) and the effects were measured at 0.5, 1.0, 1.5, 2.0, and 24 h following the application. For comparative purposes, morphine (MF, 5.0 mg/kg, *s.c.*), naloxone (NL, 3.0 mg/kg, *s.c*.), or acetylsalicylic acid (ASA, 300 mg/kg, *p.o.*) alone or together with OAO-ASA **(8**) was administered to other groups of mice. Moreover, the study included the effect of complimentary doses of ASA (0.77 mg/kg, 7.7 mg/kg, and 77.0 mg/kg, *p.o.*) to its presence in OAO-ASA **(8)** (3.0 mg/kg, 30.0 and 300.0 mg/kg, respectively). Control animals received the proper volume of 5% Tween 80 (*p.o.*) (vehicle for OAO-ASA and ASA) and 0.9% saline (*s.c.*) (vehicle for MF and NL). Using a timer, the latency to respond was observed with a hind paw lick, hind paw flick, or a jump which was measured to the nearest 0.1 s. Subsequently, the mouse was immediately removed from the hot plate and returned to its home cage. The maximum time of the response was established as 60 s.

#### Anti-inflammatory Test

Skin inflammation was induced in the right hind paw of rats by the topical application of 2 mg/paw of carrageenan dissolved in 0.2 ml of 0.9% saline solution. The rear left paw of the rats, which was used as the control, received the same volume of 0.9% saline solution. Single doses of OAO-ASA **(8)**, dissolved in 5% Tween 80, in the range of 0.3, 3.0, 30.0, and 300.0 mg/kg were given (*p.o.*) 30 min after carrageenan injection. For comparison, one group of rats was treated with the single ASA (200 mg/kg, *p.o.*) also 30 min after carrageenan administration. Moreover, the study included influence of complimentary doses of ASA (0.77 mg/kg, 7.7 mg/kg, and 77.0 mg/kg, *p.o*.) to proper doses of OAO-ASA **(8)** (3.0 mg/kg, 30.0 and 300.0 mg/kg, respectively) or the interaction between the compound **8** and ASA (200 mg/kg, *p.o.*) on anti-inflammatory activity in rats measured also 30 min after carrageenan administration. The rate of oedema of the two paws was measured at 1.5, 3.0, 6.0, and 10.0 h after carrageenan injection using a plethysmometer.

Change of rat’s paw thickness was evaluated using the following equation:

$$\underset{-}{\Delta }G=\left(L_{c}-L_{w})-(R_{c}-R_{w}\right)$$

*G* – value expressing change in paw’s thickness against baseline (before inflammation)
*L*_w_ – left paw’s thickness before carrageenan injection
*R*_w_ – right paw’s thickness before carrageenan injection
*L*_c_ – left paw’s thickness 1.5, 3.0, 6.0, or 10.0 h after carrageenan injection
*R*_c_ – right paw’s thickness 1.5, 3.0, 6.0, or 10.0 h after carrageenan injection

#### Subchronic Effect of OAO-ASA (8)

In the last step of our study, the antinociceptive activity of OAO-ASA **(8)** for mice and anti-inflammatory activity of the above conjugate **8** for rats were evaluated in selected effective dose of 30.0 mg/kg (*p.o.*) after first and repeated application once a day during four consecutive weeks.

#### Measurement of Cytokines Levels in Rats

On the next day after anti-inflammatory activity tests measurements the drug was given again and 120 min later rats were sacrificed and the blood was collected. First part of the blood was centrifuged at 4000 rpm for 15 min and the serum was separated and stored at -80°C for further biochemical measurements. These measurements were done by means of the ELISA method using commercially available kits (RayBiotech Inc.). These tests comprised recombinant cytokines from *Escherichia coli* and antibodies against rats interleukin 6 (IL-6) and Tumor Necrosis Factor alpha (TNF-α). The results were calculated based on the absorbance of complex cytokines-antibodies and concentrations were obtained from model curves according to producer protocols.

#### Influence of OAO-ASA (8) on mRNA Levels of Studied Genes

From the second part of peripheral blood of rats the mononuclear cells (MNCs) were isolated *via* a gradient centrifugation in Ficoll. From the resulting cell pellets a total RNA was isolated using TriPure Isolation Reagent (Roche) according to the manufacturer’s protocol. The integrity of RNA was visually assessed electrophoretically and spectrophotometrically (BioPhotometer Eppendorf). The 1 μg of total RNA from all samples was used for reverse transcription into complementary DNA (cDNA) using Transcriptor First Strand Synthesis Kit (Roche), according to the manufacturer’s protocol, then were stored at -20°C or used directly for quantitative real-time PCR (qRT-PCR).

The IL-6, TNF-α, Toll-like receptor 1 (TLR-1), and Toll-like receptor 3 mRNA (TLR-3 mRNA) levels were analyzed by quantitative real-time PCR using the LightCycler^®^ TM Instrument (Roche, Germany) and the LightCycler^®^ FastStart DNA Master SYBR Green I (Roche, Germany) according to the manufacturer’s instructions. All primer sequences were self-designed using Oligo 6.0 software (National Biosciences) and verified by the electrophoretical assessment and by melting curve analysis of each cDNA amplification product. A glyceraldehyde 3-phosphate dehydrogenase gene (GAPDH gene) was used as a housekeeping gene (endogenous internal standard). Standard curves were prepared from dilution of cDNA and generated from a minimum of four data points for each quantified gene. All quantitative PCR reactions were repeated twice. Data were evaluated using LightCycler Run 4.5 software (Roche Applied Science). Each PCR run included a non-template control to detect potential contamination of reagents.

### Statistical Analysis

The data were expressed as means ± SEM and the statistical comparison of results was carried out using ANOVA followed by a Duncan *post hoc* test.

## Results

### Synthesis of OAO-ASA (8)

**SCHEME 1:**
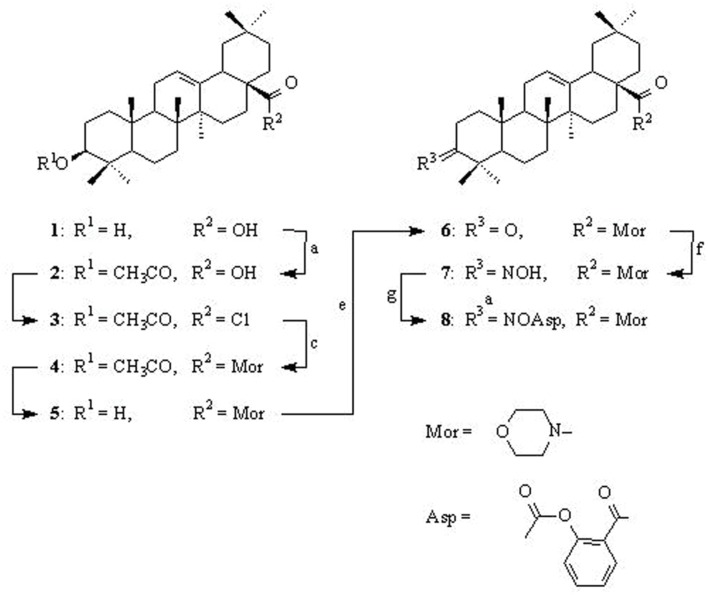
**Synthesis of conjugate OAO-ASA **(8)**.** Reagents and conditions: (a) pyridine, (CH_3_CO)_2_O, rt.; (b) SOCl_2_, rt.; (c) C_6_H_6_, morpholine, rt.; (d) C_2_H_5_OH, KOH, reflux; (e) acetone, Jones reagent, rt.; (f) C_2_H_5_OH, NH_2_OH x HCl, CH_3_COONa, reflux; (g) dioxane, ASA, DCC, rt.

The conjugate OAO-ASA **(8)** was obtained from oxime of oleanonic acid morpholide **(7)**. The oxime **7** was synthesized as it is presented in **Scheme 1** and with application of procedures known from our earlier work (Bednarczyk-Cwynar et al., 2015).

First, oleanolic acid (**1**) was acetylated with a 10-fold excess of acetic anhydride in dried pyridine. The obtained product, with a reversibly blocked hydroxyl group at C-3 position (3-*O*-acetyloleanolic acid, **(2**), was subjected to thionyl chloride action and the resulting 3-*O*-acetyloleanolic acid chloride (**3**) was transformed into 3-*O*-acetyloleanolic acid morpholide (**4**) upon the action of morpholine in dried benzene at room temperature. The obtained 3-*O*-acetylmorpholide of oleanolic acid **4** was hydrolyzed with 5% sodium hydroxide ethanolic solution with short heating. As spectral data proved, in these alkaline conditions only the ester bond at the C-3 position underwent hydrolysis to give product **5**. The morpholide of oleanolic acid with the C-3 carbonyl group (compound **6**) was obtained by oxidation of an appropriate amide with the free hydroxyl group (derivative **5**) with Jones reagent. The 3-oxoderivative **6** was subjected to reaction with hydroxylamine hydrochloride in ethanol and the resulted oxime 7 was acylated with ASA in dioxane in the presence of *N*,*N*-DCC (Bednarczyk-Cwynar et al., 2012; Kaminskyy et al., 2012) to give the conjugate OAO-ASA **(8)**.

### Acute Toxicity Test

It was found that acute administration of OAO-ASA **(8)** to male mice (*n* = 5) at the dose of 2.0 g/kg b.w. (*p.o.*), did not show any mortality or toxic effects during the next 21 days.

### Locomotor Activity Test

**FIGURE 1:**
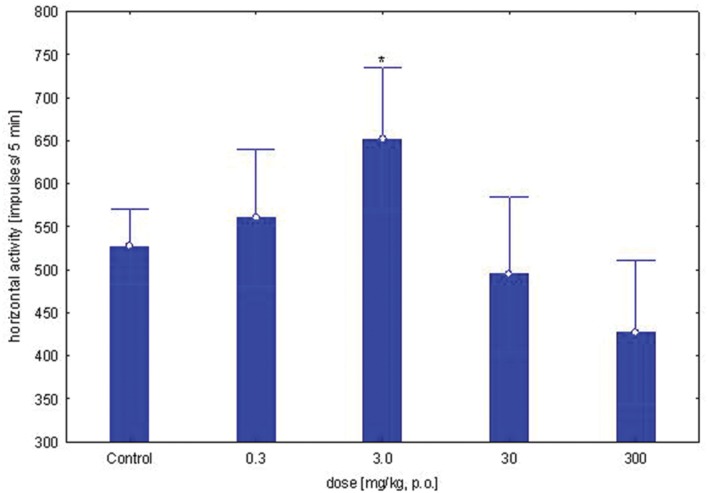
**Dose-dependent effect of OAO-ASA **(8)** on horizontal locomotor activity in mice.** Number of mice = 9–10 in each group, data are mean ± SEM, control = mice treated with 5% Tween 80, ^∗^ = vs. Control, *p* < 0.05.

The study was performed using five groups of mice (*n* = 9–10 in each group) treated with OAO-ASA **(8)** in doses of 0.3, 3.0, 30.0, and 300.0 mg/kg (*p.o.*) and control (5% Tween 80). One-way ANOVA analysis of variance has presented that single administration of the compound **8** in the above range of doses has not shown a statistically significant effect on horizontal locomotor activity in mice [*F*(4,56) = 3.90; *p* = 0.007]. The impact of the individual doses of OAO-ASA **(8)** on horizontal locomotor activity revealed that only a dose of 3.0 mg/kg has increased mobility of the animals during horizontal activity test (*p* < 0.05). In addition, a decrease in activity was observed after administration of 300.0 mg/kg of this compound **8**, but the average value obtained was not significantly different compared to the control values (*p* > 0.05; **Figure 1**).

### Analgesic Activity – Hot-Plate Test

#### Dose-Dependent Study

**FIGURE 2:**
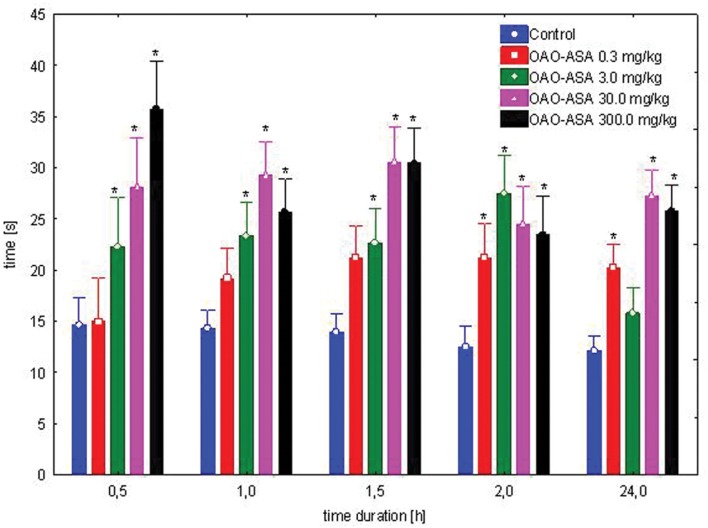
**Dose-dependent analgesic effect of OAO-ASA **(8)** in mice.** Number of mice = 9–10 in each group, data are mean ± SEM, control = mice treated with 5% Tween 80, ^∗^ = vs. proper Control, *p* < 0.05.

The study was performed using five groups of mice (*n* = 9–10 in each group) treated with OAO-ASA **(8)** in doses of 0.3, 3.0, 30.0, and 300.0 mg/kg and control (5% Tween 80). Administration of conjugate **8** has shown a statistically significant analgesic activity in the hot-plate test performed on mice [ANOVA main effect *F*(4,56) = 53.3, *p* = 0.000] and has indicated a significant effect of time on this type of activity [ANOVA, *F*(4,224) = 4.46; *p* = 0.002]. The interaction between group and time effect also has shown high significance [ANOVA II: *F*(16,224) = 4.96; *p* = 0.000]. Based on further analysis performed by a *post hoc* test, it was found that there are significant differences in inhibition of the pain response by OAO-ASA **(8)** after thermal stimuli (**Figure 2**). The peak of analgesic effect of compound **8** was observed after administration of the compound at a dose of 300.0 mg/kg after 30 min. It was also found that the analgesic action of conjugate **8** appeared after using the lowest tested dose (0.3 mg/kg) after 2 h (*p* < 0.05) and this particular effect was maintained up to 24 h. However, OAO-ASA **(8)** given at a dose of 3.0 mg/kg was active at 0.5 h after starting the test, and the strongest activity was observed after 2 h, but the effect was not statistically significant at 24th hour of the experiment. For the analyzed compound **8** in doses of 30.0 and 300.0 mg/kg, the analgesic effect was visible throughout the course of the study and continued up to 24 h of the experiment compared to the control values (*p* < 0.01).

#### Analgesic Effect of Complimentary Doses of ASA

**FIGURE 3:**
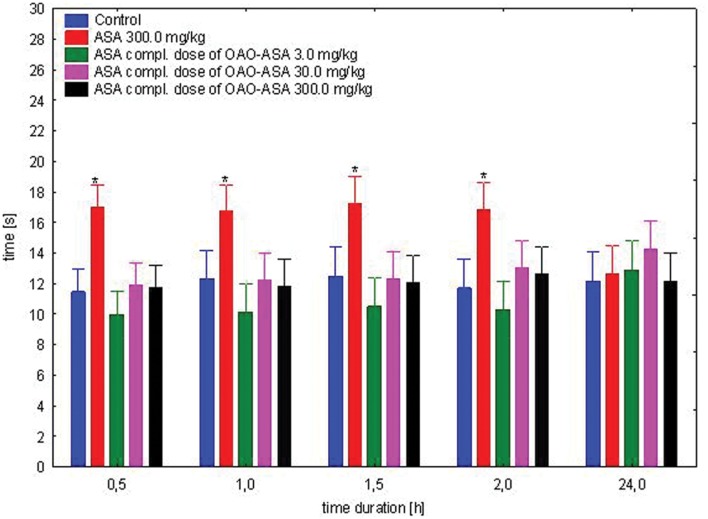
**Effect of complimentary dose of ASA on analgesic action of OAO-ASA **(8)** in mice.** Number of mice = 9–10 in each group, control = mice treated with 5% Tween 80, data are mean ± SEM, ^∗^ = vs. proper Control, *p* < 0.05.

The study was performed using four groups of mice (*n* = 9–10 in each group) treated intragastrically with ASA (0.77, 7.7, and 77.0 mg/kg) in complimentary doses to OAO-ASA **(8)** of 3.0, 30.0, and 300.0 mg/kg (*p.o.*); referent dose of ASA in 300.0 mg/kg and control (5% Tween 80). Total variability in the tested doses for the analgesic effect of complimentary doses of ASA has been noted [ANOVA: *F*(3,35) = 9.04; *p* = 0.000], but has not detected a statistically significant effect of time on the antinociceptive effect in the analyzed experimental system [ANOVA: *F*(4,140) = 0.812; *p* = 0.519]. Analyzing the impact of group and time effects, the occurrence of variation between the values were obtained [ANOVA II: Interaction *F*(12,140) = 2.95; *p* = 0.001]. Further analysis of ASA complementary doses to conjugate **8** for an analgesic effect in the mice hot-plate assay has indicated that only administration of ASA at the dose of 300.0 mg/kg has shown significant analgesic activity in comparison to the control group (**Figure 3**). The effect of ASA at this dose was visible from 0.5 to 2 h after the application, but no analgesic effect was observed after 24 h. Also, no significant differences for the ASA stated in all complementary doses of ASA for the content of the analyzed compound **8** relative to control values were observed.

#### Interaction of OAO-ASA (8) with ASA

**FIGURE 4:**
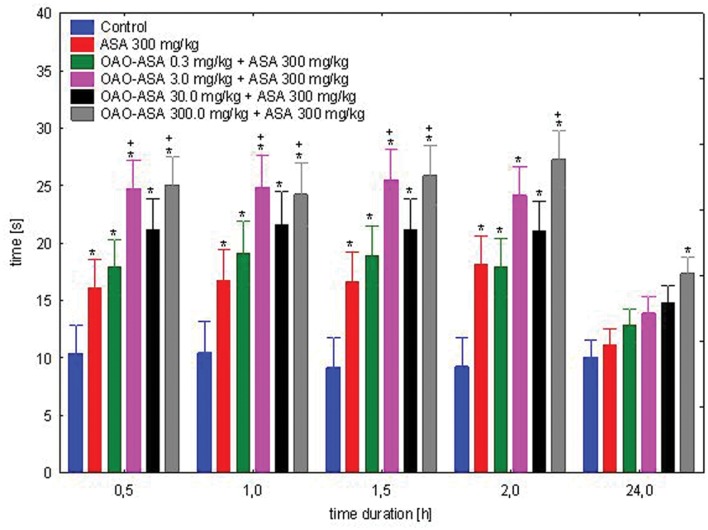
**Effect of OAO-ASA **(8)** on antinociceptive activity of ASA (300.0 mg/kg *p.o.*) in mice.** Number of mice = 9–10 in each group, data are mean ± SEM, control = mice treated with 5% Tween 80, ^∗^ = vs. proper Control, *p* < 0.05, + = vs. ASA 300 mg/kg, *p.o.*, *p* < 0.05.

The study was performed using 6 groups of mice (*n* = 9–10 in each group) treated with OAO-ASA **(8)** in doses of 0.3, 3.0, 30.0, and 300.0 mg/kg (*p.o.*) and ASA in the dose of 300.0 mg/kg, *p.o.*; for control purposes the effect of ASA only in the dose of 300.0 mg/kg was also measured in comparison to control rats (5% Tween 80). When analyzing the effect of the co-administration of OAO-ASA **(8)** and ASA for antinociceptive activity in the hot-plate assay, the existence of a general statistical variation in experimental system has been confirmed [ANOVA, effect of group, *F*(5,53) = 35.7; *p* = 0.000], as well as the significant effect of time on the antinociceptive activity [ANOVA: *F*(4,212) = 51.5; *p* = 0.000]. The interaction of these two effects (i.e., the effect of group and time) showed the existence of significant differences between these two parameters [ANOVA: interaction *F*(20,212) = 3.827; *p* = 0.000]. *Post hoc* analysis has shown the presence of the antinociceptive effect of ASA given at a dose of 300.0 mg/kg in the range of 0.5–2 h from the start of the study (*p* < 0.05), but after 24 h, there was no significant difference in comparison to the control value (**Figure 4**). For the combined administration of the examined substance OAO-ASA **(8)** and ASA over the range of doses used, the significant differences between obtained values and control at the time 0.5, 1, 1.5, and 2 h were noted. After 24 h, the effect after co-administration of ASA and the compound **8** at doses of 0.3–30.0 mg/kg was not significantly different from that of the control group administered with vehicle alone, only in the highest dose of the OAO-ASA **(8)** co-administered with ASA has differed significantly from control values (*p* < 0.05). It has been noted that conjugate **8** in a dose of 300.0 mg/kg significantly increased the strength and prolonged the duration of antinociceptive action of ASA in each of the time points tested; while at a dose of 3.0 mg/kg the increased potency of ASA after combined administration was seen only in 0.5, 1, and 1.5 h of the experiment.

#### Interaction of OAO-ASA (8) with MF

**FIGURE 5:**
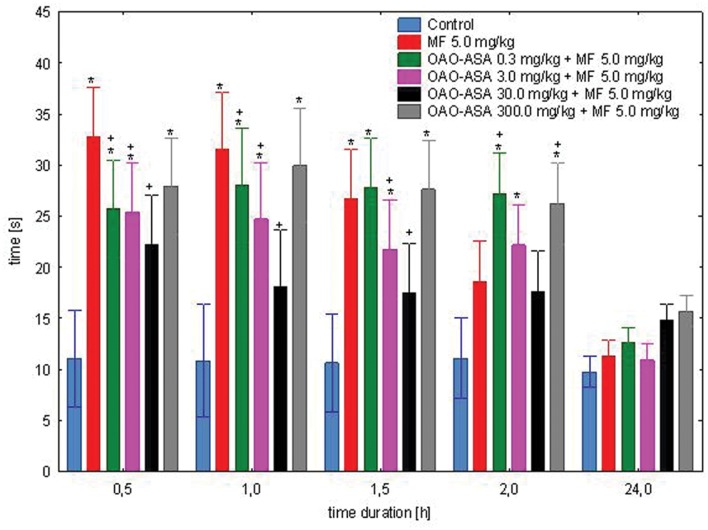
**Effect of OAO-ASA **(8)** on analgesic activity of MF (5.0 mg/kg, *s.c.*) in mice.** Number of mice = 9–10 in each group, data are mean ± SEM, control = mice treated with 5% Tween 80, 0.9% NaCl, ^∗^ = vs. proper Control, *p* < 0.05, + = *vs.* MF 5.0 mg/kg, *s.c.*, *p* < 0.05.

The study was performed using six groups of mice (*n* = 9–10 in each group) treated with OAO-ASA **(8)** in doses of 0.3, 3.0, 30.0, and 300.0 mg/kg (*p.o.*) and MF in a dose of 5.0 mg/kg (*s.c.*); for comparative purposes MF in a dose of 5.0 mg/kg and proper control group were used (5% Tween 80; 0.9% NaCl). Study on the combined administration of the examined compound **8** and MF was able to show the overall variability between means [ANOVA, main effect, *F*(5,54) = 10.2; *p* = 0.000], and highlighted the importance of the effect of time on the course of the analgesic effect [ANOVA, effect of time, *F*(4,216) = 64.6; *p* = 0.000]. The interaction of these two effects (i.e., the effect of group and time) showed the existence of significant differences between these two parameters [ANOVA, interaction *F*(20,216) = 6.02; *p* = 0.000]. *Post hoc* analysis revealed the analgesic effect of MF after 0.5, 1, and 1.5 h of the test when compared with the control group (*p* < 0.05); whereas after 2 and 24 h showed no significant effects (**Figure 5**). Co-administration of MF and OAO-ASA **(8)** in a dose of 0.3 mg/kg has shown a significant analgesic effect that lasted for 2 h from the start of the test, and in 0.5 h of the test significantly increased the analgesic activity of MF. For the compound **8** in the doses of 3.0 and 300.0 mg/kg combined with MF, the analgesic effect was shown in all tested intervals except for 24 h. The least effective was administration of MF with conjugate **8** at a dose of 30.0 mg/kg, where no significant antinociceptive activity was shown in any of the time points tested. Moreover, it has been found that the combination of the OAO-ASA **(8)** in a dose of 3.0 mg/kg with MF significantly lowered pain response at 0.5 and 1 h when compared with MF. There were no significant differences as compared to combined administration to the mean values of MF at other points of time.

#### Interaction of OAO-ASA (8) with NL

**FIGURE 6:**
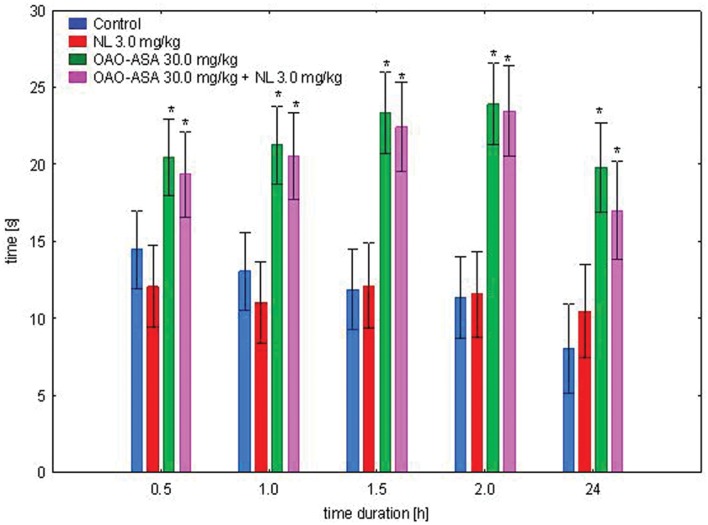
**Effect of OAO-ASA **(8)** on analgesic activity of NL (3.0 mg/kg, *s.c.*) in mice.** Number of mice = 9–10 in each group, data are mean ± SEM, control = rats treated with 5% Tween 80; 0.9% NaCl, ^∗^ = vs. proper Control, *p* < 0.05.

The study was performed using four groups of mice (*n* = 9–10 in each group) treated with OAO-ASA **(8)** in a dose of 30.0 mg/kg without and with NL in a dose of 3.0 mg/kg (*s.c.*); NL in 3.0 mg/kg alone and control group (5% Tween 80; 0.9% NaCl). The experimental system demonstrated the existence of variation between the means [ANOVA main effect, *F*(3,37) = 31.9; *p* = 0.000] and indicated the significance of the effect of time [ANOVA effect of time *F*(4,148) = 8.38, *p* = 0.000]. Analysis of the interaction of both factors studied in this test indicated the importance of the action of both effects [ANOVA, interaction *F*(12,148) = 2.14; *p* = 0.018]. Further detailed analysis allowed to determine that administration of NL in all time points did not show significant analgesic activity compared to the corresponding control values (**Figure 6**). It was found that for the entire test time interval, OAO-ASA **(8)** given with and without NL resulted in statistically significant values when compared with corresponding control values (*p* ≤ 0.01). No significant differences were detected in addition to the analgesic effect between the group receiving conjugate **8** and the group receiving the combination of the tested compound **8** with NL (*p* > 0.05).

### Anti-inflammatory Effects

#### Dose-Dependent Study

**FIGURE 7:**
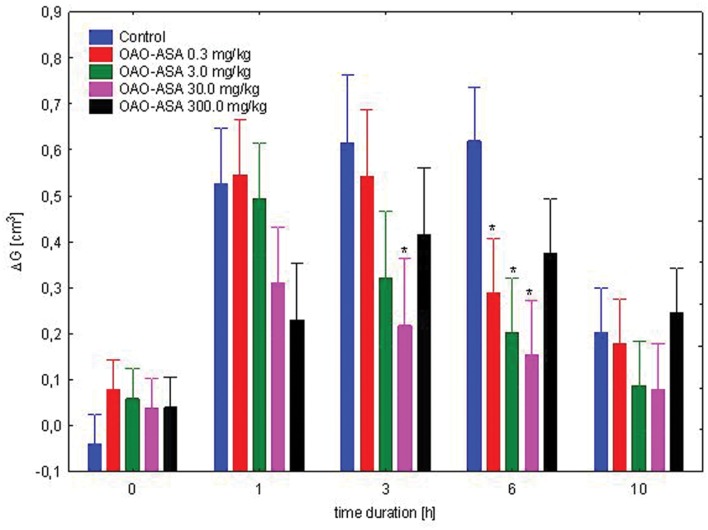
**Dose-dependent anti-inflammatory effect of OAO-ASA **(8)** in rats.** Number of rats = 8 in each group, data are mean ± SEM, control = rats treated with 5% Tween 80, ^∗^ = vs. proper Control, *p* < 0.05.

The study was performed using five groups of rats (*n* = 8 in each group) treated with OAO-ASA **(8)** in doses of 0.3, 3.0, 30.0, and 300.0 mg/kg and control (5% Tween 80). It was noted that administration of the compound **8** showed a statistically significant anti-inflammatory activity in the experimental system used [ANOVA, the main effect, *F*(4,35) = 4.48; *p* = 0.000; effect of time *F*(4,140) = 81.5, *p* = 0.000]. The interaction of both factors indicated the importance of the action of both [ANOVA, interaction *F*(16,140) = 6.91, *p* = 0.000]. Further *post hoc* analysis allowed to determine that the anti-inflammatory effect in relation to the control group. This was characterized by administration of the analyzed compound (triterpene **8**) at a dose of 0.3 mg/kg after 6 h (*p* < 0.05), for a dose of 3.0 mg/kg was of weak significance (*p* = 0.051) at the third hour after start of the test, but statistically significant effect was observed at sixth hour of the study (*p* ≤ 0.05), for a dose of 30.0 mg/kg, this effect already appeared in third hour (*p* < 0.05) and was still visible after 6 h (*p* < 0.01) when compared with the corresponding control values (**Figure 7**).

#### Anti-inflammatory Effect of Complimentary Doses of ASA

**FIGURE 8:**
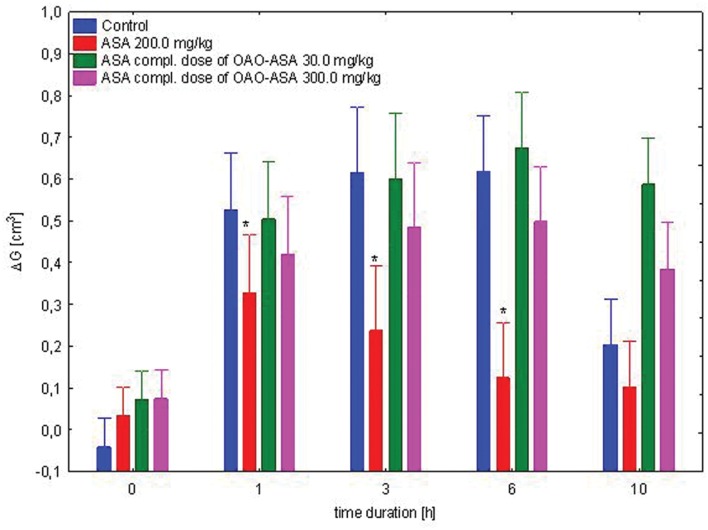
**Effect of complimentary dose of ASA on anti-inflammatory activity of OAO-ASA **(8)** in rats.** Number of rats = 8 in each group, data are mean ± SEM; control = rats treated with 5% Tween 80, ^∗^ = vs. proper Control, *p* < 0.05.

The study was performed using four groups of rats (*n* = 8 in each group) treated with ASA (7.7 and 77.0 mg/kg, *p.o.*) in complimentary doses to OAO-ASA **(8)**: 30.0 and 300.0 mg/kg; ASA in a referent dose of 200.0 mg/kg and control (5% Tween 80). Analysis of variance used during the experiment has indicated a statistically significant anti-inflammatory effect either for a group [ANOVA effect of group *F*(3,28) = 8.99; *p* = 0.000] and for time as a factor associated with anti-inflammatory effect in this experimental system [ANOVA: *F*(4,112) = 68.6; *p* = 0.000]. It has been shown that the interaction of both of these factors are also statistically significant [ANOVA, interaction *F*(12,122) = 6.806; *p* = 0.000]. Detailed analysis of the *post hoc* test showed that only ASA significantly reduced the carrageenan-induced edema at first, third hour (*p* < 0.05) and sixth hour of observation (*p* < 0.01) when compared with respective control values (**Figure 8**).

#### Interaction of OAO-ASA (8) with ASA

**FIGURE 9:**
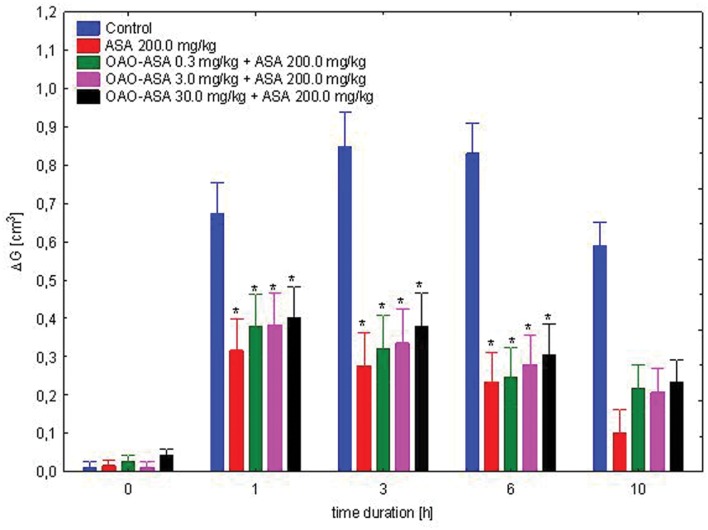
**Effect of OAO-ASA **(8)** on anti-inflammatory activity of ASA (200.0 mg/kg, *p.o.*) in rats.** Number of rats = 8 in each group, data are mean ± SEM, control = rats treated with 5% Tween 80, ^∗^ = vs. proper Control, *p* < 0.05.

The study was performed using five groups of rats (*n* = 8 in each group) treated with OAO-ASA **(8)** in doses of 0.3, 3.0, and 30.0 mg/kg and ASA in a dose of 200.0 mg/kg; referent single dose of ASA in 200.0 mg/kg and control (5% Tween 80). The following experimental system was found to have significant variability in the results of the performance of the combined administration of ASA and OAO-ASA **(8)** in rats [ANOVA, main effect, *F*(4,35) = 34.2; *p* = 0.000]. It also indicated the importance of activity of time after the administration of carrageenan and the compounds [ANOVA, effect of time *F*(4,140) = 284.3; *p* = 0.000]. Analysis of the interaction of both factors pointed at the significance of the action of both [ANOVA II interaction *F*(16,140) = 20.8; *p* = 0.000]. *Post hoc* analysis showed that both, the single ASA administration and conjugate **8** with ASA demonstrated the anti-inflammatory effect in a range 1–10 h when compared with the proper control values (*p* < 0.01; **Figure 9**). It was also shown that the effects did not differ between the values for combined administration of OAO-ASA **(8)** in all doses with ASA and the single ASA administration (*p* > 0.05).

#### Subchronic Analgesic and Anti-inflammatory Activity of OAO-ASA (8)

**FIGURE 10:**
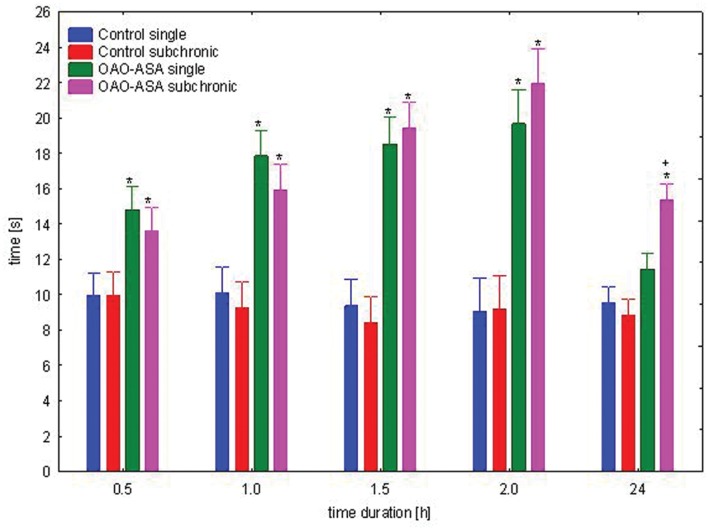
**Comparison of analgesic activity of OAO-ASA **(8)** after single and subchronic administration in mice.** OAO-ASA **(8)** treatment with a dose of 30 mg/kg, *p.o.* Number of mice = 10 in each group, data are mean ± SEM, control – mice treated with 5% Tween 80, ^∗^ = vs. proper Control, *p* < 0.05, + = vs. proper OAO-ASA **(8)** single, *p* < 0.05.

The study was performed using two groups of mice (*n* = 10 in each group) treated with OAO-ASA **(8)** in a dose of 30.0 mg/kg and analgesic activity was assessed after first and after 28 applications of triterpene **8**. Analysis of variance used during the experiment has indicated a statistically significant analgesic effect either for a group [ANOVA, main effect, *F*(3,36) = 76.2; *p* = 0.000] and for time as a factor associated with this activity in the experimental model [ANOVA, effect of time, *F*(4,144) = 25.0; *p* = 0.000]. *Post hoc* analysis showed that both, after single and subchronic administration OAO-ASA **(8)** produced significant analgesic activity (*p* < 0.05) in time range of 0.5–2 h when compared with proper control values (**Figure 10**). However, due to the fact that obtained values did not differ between each other for the time, therefore an occurrence of tolerance for analgesic activity might be excluded.

**FIGURE 11:**
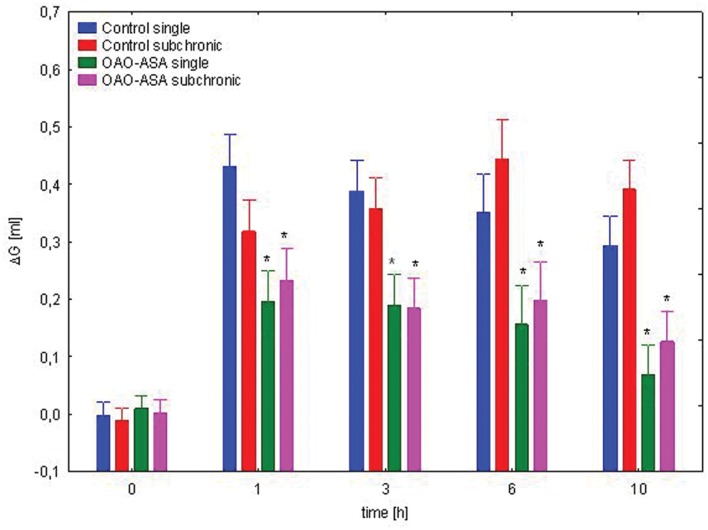
**Comparison of anti-inflammatory activity of OAO-ASA **(8)** after single and subchronic administration in rats.** OAO-ASA **(8)** treatment with a dose of 30 mg/kg, *p.o.* Number of rats = 10 in each group, data are mean ± SEM, control = rats treated with 5% Tween 80, ^∗^ = vs. proper Control, *p* < 0.05.

The study for the assessment of the effect of subchronic treatment on anti-inflammatory activity of OAO-ASA **(8)** was performed using two groups of rats (*n* = 10 in each group) treated with the triterpenic conjugate **8** in a dose of 30.0 mg/kg and the activity was measured after first and after 28 applications of OAO-ASA **(8)**. Analysis of variance used during the experiment has indicated a statistically significant analgesic effect either for a group [ANOVA, main effect, *F*(3,36) = 23.3; *p* = 0.000] and for time as a factor associated with this activity in the experimental model [ANOVA, effect of time, *F*(4,144) = 168.1; *p* = 0.000]. *Post hoc* analysis showed that both the single and subchronic conjugate **8** administration significantly reduced the carrageenan-induced edema in the range of 1–10 h of observation (*p* < 0.05) when compared with the proper control values (**Figure 11**). Similarly, as for the analgesic activity of OAO-ASA **(8)** there were no differences between the values for single and subchronic treatments in the time points (1–10 h), therefore effect of tolerance for the anti-inflammatory activity should be excluded.

#### Peripheral Cytokines Concentrations and mRNA Expression Changes

A one-way ANOVA revealed significant difference between the groups of rats in the IL-6 mRNA levels [*F*(1,18) = 11.7; *p* < 0.001; **Table 1**]. Subchronic treatment with OAO-ASA **(8)** led to a significant decrease of the cytokine concentration compared to the control group. On the contrary, the one-way ANOVA showed insignificant difference between the groups in the TNF-α levels [*F*(1,17) = 0.953; *p* > 0.05; **Table 1**], however, there was a tendency to lower the cytokine concentration in rats after OAO-ASA **(8)** treatment. Statistical analysis of IL-6 mRNA expression showed significant difference between the groups [ANOVA: *F*(1,15) = 4.90; *p* < 0.05; **Table 1**] and the subchronic OAO-ASA **(8)** administration lowered the mRNA expression of this cytokine. Similarly, the ANOVA revealed significant differences between the groups of rats in the TNF-α mRNA expression [*F*(1,18) = 14.1, *p* < 0.01; **Table 1**], but OAO-ASA **(8)** administration caused a strong increase of the measured parameter. Additionally, effect of OAO-ASA **(8)** on the mRNA expression of TRL-1 and TLR-3 was measured. It was found that there was a significant difference between the groups for the TRL-1 mRNA expression [*F*(1,18) = 5.14; *p* < 0.05; **Table 1**] and OAO-ASA **(8)** administration showed an increased transcription level compared to the control group. On the contrary, a one-way ANOVA revealed insignificant difference between the groups in the TLR-3 mRNA expression [*F*(1,18) = 0.117; *p* > 0.05; **Table 1**].

**Table 1:** Interleukin 6 (IL-6) and Tumor Necrosis Factor-alpha (TNF-α) serum levels and IL-6, TNF-α, TLR-1, and TLR-3 mRNA expression of rats subchronically treated with OAO-ASA **(8)**.

Group^n^	Serum concentration (pg/ml)		mRNA expression^∗^ (%)			
		
	IL-6	TNF-α	IL-6	TNF-α	TLR-1	TLR-3
Control	55.4 ± 1.6	1494 ± 332	100 ± 15	100 ± 7	100 ± 10	100 ± 13
OAO-ASA (8)	9.3 ± 0.8^∗∗^	1099 ± 201	60 ± 5^∗∗^	11 ± 29^∗∗^	138 ± 13^∗∗^	108 ± 19


*TLR-1, TLR-3 = Toll-like receptor 1, Toll-like receptor 3, values are means ± SEM, n = number of animals: 10, ^∗^ = values expressed as a ratio: the gene/GAPDH, control = rats treated with 5% Tween 80, OAO-ASA **(8)** = rats treated with OAO-ASA **(8)** (dissolved in 5% Tween 80) in the dose of 30 mg/kg (*p.o.*) for 4 weeks, ^∗∗^ = vs. control, *p* < 0.05.*

## Discussion

The triterpenic conjugate of oxime of oleanolic acid morpholide **(7)** and ASA, named as OAO-ASA **(8)** was obtained in a simple reaction of the above oxime **(7)** and ASA in dried dioxane in the presence of *N*,*N*-DCC as condensing agent. Stirring of these three reagents in dioxane at room temperature led only to one product, as TLC control proved. The received crude product **8** have not formed crystals and was purified by re-precipitation with water from ethanolic solution.

The analysis of ^1^H and ^13^C NMR spectra of the obtained product OAO-ASA **(8)** proved structure of this compound. Signals derived from aromating ring of ASA were observed at δ 7.97 (1H, dd), 7.57 (1H, td), 7.32 (1H, td), and 7.13 (1H, dt) ppm. Signal from the acetoxy function of ASA within conjugate **8** was observed as singlet at 2.34 ppm. The morpholine ring protons of OAO-ASA **(8)** gave strong multiplet that was present at 3.70–3.58 ppm (8H). In ^13^C NMR seven characteristic quaternary signals were observed, at δ176.3, 175.1, 169.6, 162.0, 150.6, 144.8, and 122.8 ppm. First and second of them (176.3 and 175.1 ppm) were attributed to the C-3 and C-28 atoms within the molecule of OAO-ASA **(8)** (Bednarczyk-Cwynar et al., 2015), respectively; third of them, present at δ 169.6 ppm derived from acetoxyl (CH_3_COO–) within ASA; the next one (at δ 162.0 ppm) pointed at the carboxyl function linked to nitrogen atom (–COON=). The two further quaternary signals, present at 150.6 and 122.8 ppm, were assigned to ASA ring and the last quaternary carbon, observed at 144.8 ppm, derived from C-13 atom (Bednarczyk-Cwynar et al., 2015). The remaining four tertiary aromatic signals were observed at δ 133.7, 131.2, 125.9, and 124.0 ppm. The morpholine ring signals were present at typical values of chemical shifts: δ 66.9 × 2, 47.4 and 47.2 ppm (Bednarczyk-Cwynar et al., 2015).

In pharmacological part of our research, at first, the acute toxicity of OAO-ASA **(8)** was assessed. The results allowed maintaining that the analyzed compound **8** shows low toxicity (LD_50_ > 2 g/kg) and belongs to Toxicity Class 1 (data not shown). This is in agreement with the results of other studies that this class of compounds possesses rather low toxicity (Singh et al., 1992; Liu, 1995; Bednarczyk-Cwynar et al., 2012).

In the next part of the study, we decided to check whether OAO-ASA **(8)** can affect locomotor activity of mice, since an eventual sedation (expressed by lowering of animals’ activity) can give false positive results in hot-plate experiment. The test is sometimes performed also in order to answer whether or not a new substance has an impact on the behavior of animals coupled with some aspects of cognition and fear (Piazza et al., 1989; Cools and Gingras, 1998). It was found that OAO-ASA **(8)** only in the dose of 3.0 mg/kg increased the horizontal locomotor activity, whereas, in the rest of doses, it did not affect this paradigm, therefore the risk of sedation can be excluded from OAO-ASA **(8)** pharmacological profile. Generally the results are in line with observations coupled with pharmacological profile of many triterpenes obtained by other authors (Oliveira et al., 2005; Soldi et al., 2008; Marcon et al., 2009; Bednarczyk-Cwynar et al., 2012).

It was observed that OAO-ASA **(8)** exhibited antinociceptive effect in the applied model using the hot-plate test, which represents a tool for the study of supraspinal pain. The test is generally used for centrally acting analgesic drugs such as morphine, whereas for the peripherally acting analgesics is rather ineffective (Matthes et al., 1996; Milano et al., 2008). Moreover, the test was performed in several other studies of derivatives of oleanolic acid (Jung et al., 2005; Otuki et al., 2005a; Bednarczyk-Cwynar et al., 2012). Since there are opinions that oleanolic acid and its some derivatives produce the antinociceptive effect via opioid mechanism of action (Maia et al., 2006b; Park et al., 2013), therefore the next stage of the experiment was to test a possible interaction with opioid receptor agonist MF and pure antagonist of opioid receptors NL. Suggesting from our previous studies MF was selected for this purpose in a dose of 5.0 mg/kg administered subcutaneously (Siekierkowska et al., 2007) and was in the range of doses used by other investigators (Maldonado et al., 1998; Sammons et al., 2000; Tsai et al., 2000). In the same manner NL was administered, as an antagonist of the opioid receptors in a dose of 3.0 mg/kg (Bodnar et al., 1978; Mena et al., 1996; Li et al., 2004; McGaraughty et al., 2005). The study showed the interaction between the tested OAO-ASA **(8)** and opioid agonist, since especially in the dose of 30.0 mg/kg the conjugate **8** significantly decreased central effects of analgesia induced by opioid, but there was no effect on test values in mice after a combined application with NL. The results rule rather analgesic activity of the tested compound **8** through the mechanism of opioid proposed by some authors (Maia et al., 2006b; Park et al., 2013), related to the increased release of endogenous opioid or agonist effects on opioid receptors. Therefore, it is in line with the results of Otuki et al. (2005a) showing that some oleanolic acid derivatives did not express centrally mediated analgesic activity.

The antinociceptive analgesic activity of the compound **8** after single administration was maintained up to 24 h from the start of the test on the same level especially in the dose of 30.0 mg/kg, but it lasted longer than the effect of reference substance ASA (300 mg/kg, *p.o.*) in the same test. It should be stressed that ASA also exhibits an antinociceptive effect in the hot-plate test in rodents (Heidari et al., 2004). It was observed the strongest activity of the compound **8** (*t*_max_) for 30 min of the administration. However, the possible mechanism of the compound **8** for its antinociceptive activity was rather similar to ASA action, since testing the compound OAO-ASA **(8)** interaction with ASA, only in the lowest dose there was no synergistic effect on the activity of ASA model used, while in the rest of doses a significant effect of synergistic interaction with ASA was found.

In order to determine the possible contribution of ASA activity released from the test compound **8** in rodents, the use of ASA in complimentary doses to its presence in OAO-ASA was performed. The conducted experiments generally indicated that ASA in complementary doses to the doses used for OAO-ASA **(8)** did not affect the analgesic activity. It shows that rather the whole molecule is responsible for the analgesic effect of the tested compound **8**, however, it cannot be excluded that the summarizing effect is produced by ASA released from the compound **8** and the rest of triterpene derivative. To test this hypothesis more detailed study should be performed.

In a next step, we decided to evaluate the dosage-dependent effects of the OAO-ASA **(8)** for the its anti-inflammatory activity in the carrageenan test. The results has proven to be significant relative to the control rats, which enabled the determination of dose-effect. The strongest effect of OAO-ASA **(8)** was observed in the dose of 30.0 mg/kg both for the analysis of individual time points, and the same dose-effect was described shape of an inverted “U.” The results are in line with studies on anti-inflammatory activity of some oleanolic acid derivatives (Otuki et al., 2005b; Medeiros et al., 2007; Chung et al., 2008; Preethi et al., 2009). It can speculate that compound **8** probably inhibits COX-2 activity and production of PGE2. It is due to the fact that after 60 min from carrageenan administration the prostaglandin phase of inflammation appears (Al-Majed et al., 2003). Moreover, from many studies concerning mechanism of anti-inflammatory action of oleanolic acid derivatives it is known that the effects are on transcriptive level via NF-κB which is coupled with COX-2 inhibition (Medeiros et al., 2007; Chung et al., 2008; Martinez-Gonzales et al., 2008; Nihiem et al., 2011; Jung et al., 2013; Kim et al., 2014).

In order to determine the possible contribution of ASA anti-inflammatory activity released from the test compound **8** in rats, the use of ASA in complimentary doses to its presence in OAO-ASA was performed. It shows that rather the whole molecule is responsible for the anti-inflammatory effect of the tested compound **8**, however, it cannot be excluded that the summarizing effect is produced by ASA released from the compound **8** and the rest of triterpene derivative. To test this hypothesis more detailed study should be performed. However, the OAO-ASA **(8)** anti-inflammatory mechanism of action is probably more complicated since there was no synergistic interaction between the compound **8** and ASA.

Basing on the obtained results of the evaluation of analgesic activity, with the assessment of locomotor activity, as well as the evaluation of anti-inflammatory action, it was concluded that the best activity of OAO-ASA **(8)** was obtained in a dose of 30.0 mg/kg which qualified for further study concerning the phenomenon of tolerance to analgesic and anti-inflammatory activities. Both the analgesic action and anti-inflammatory effects did not find any statistically significant differences in the activity of triterpene **8** after single or subchronic administration. That meant that the scheduled test compound OAO-ASA **(8)** did not show tolerance to suppress pain and swelling, at least during the 4-week treatment. The results are in line with the observation found by Ferreira et al. (2000) that the methanolic extract from *Epidendrum mosenii* contained two triterpenes (pholidotin and 24-methylenecycloartenol) given daily for to seven consecutive days did not develop tolerance to its antinociceptive activity in the hot-plate test.

After 4 weeks of treatment with OAO-ASA **(8)** the concentration of selected proinflammatory cytokines, i.e., TNF-α and IL-6 in serum as well as the mRNA expression for the TNF- α, IL-6, TLR-1, and TLR-3 genes in lymphocytes were performed. The study of TLR-3 mRNA expression was chosen because of the widely speculations among researchers concerning a significance of these receptors in the development of subchronic pain (Qi et al., 2011; Qian et al., 2011; Liu et al., 2012). It is known that TLR-3 is localized in astrocytes, microglia, dendritic cells and blood cells, including B lymphocytes, while TLR-1 is on surface of phagocytic cells (macrophages) and monocytes (Hornung et al., 2002; Akira, 2003; Nicotra et al., 2012). Although the main role of TLR-1 is to response to bacterial infection and participation in the resistance (Peng and Zhang, 2014), we decided to check its possible effect of subchronic OAO-ASA **(8)** administration on the carrageenan-induced inflammation. It is known that the carrageenan induces induce an inflammatory response via toll-like receptors especially TLR-4 (Bhattacharyya et al., 2008). Moreover, there is report that some triterpenes (i.e., glycyrrhizin) strongly attenuated inflammatory responses induced by TLR-3 ligands (Schrofelbauer et al., 2009).

The resulting phenomena shows that the profile of OAO-ASA **(8)** expresses its analgesic activity rather not *via* opioid mechanisms, and it is probably a component of its anti-inflammatory effect. It appears that it correlates in part with inhibition of proinflammatory effects of IL-6 (Scheller et al., 2011), in response to induced inflammation, both at the protein level as the level of mRNA expression of this cytokine. However, the anti-inflammatory properties of the analyzed compound (OAO-ASA, **8**) are not clear because although there was a tendency to lower TNF-α level, however, at the level of mRNA expression of the cytokine, opposite results were obtained. Similarly, it should be interpreted impact of the analyzed compound **8** on the expression of TLR-1, which was elevated due to the administration of the compound **8** without affecting the TLR-3 gene expression.

Although sometimes a lack of correlation between the results at the formation of the protein and transcriptional occurs and reflects the complex interaction at the level of transcription and translation of particular substance of natural origin (Ożarowski et al., 2013, 2015; Mikołajczak et al., 2015), clearly establish the profile of anti-inflammatory OAO-ASA **(8)** and the mechanisms responsible for this is not possible on the basis of the obtained results.

## Conclusion

The obtained results regarding the analgesic and anti-inflammatory activity of new conjugate of oleanolic acid derivative and acetylsalicylic acid (OAO-ASA, **8**) is interesting, but for explanation of its mechanism of action, more detailed studies are necessary.

## Author Contributions

BB-C: Planning of chemical part of research, synthesis of all intermediates and OAO-ASA, manuscript preparation; NW, MS, EK, AB, JB-W, PM: planning of pharmacological part of research and test performing; PM: manuscript preparation, approval of final version of manuscript; LZ: approval of final version of manuscript. All authors were involved in results developments and result discussion. All authors approved the final manuscript.

## Conflict of Interest Statement

The authors declare that the research was conducted in the absence of any commercial or financial relationships that could be construed as a potential conflict of interest.
